# Bioinformatics identification of ferroptosis-related genes and therapeutic drugs in rheumatoid arthritis

**DOI:** 10.3389/fmed.2023.1192153

**Published:** 2023-07-13

**Authors:** Xianbin Li, Andong He, Yue Liu, Yuye Huang, Xueli Zhang

**Affiliations:** ^1^Institute of Computational Science and Technology, Guangzhou University, Guangzhou, Guangdong, China; ^2^School of Computer Science of Information Technology, Qiannan Normal University for Nationalities, Duyun, Guizhou, China; ^3^Department of Respiratory and Critical Medicine, Ningbo First Hospital, Ningbo, Zhejiang, China; ^4^Department of Medical Technology, Zhengzhou Railway Vocational and Technical College, Zhengzhou, Henan, China

**Keywords:** rheumatoid arthritis, ferroptosis, bioinformatics analysis, therapeutic targets, pathway analysis

## Abstract

**Introduction:**

Rheumatoid arthritis (RA) is a chronic immune disease characterized by synovial inflammation and bone destruction, with a largely unclear etiology. Evidence has indicated that ferroptosis may play an increasingly important role in the onset and development of RA. However, ferroptosis-related genes are still largely unexplored in RA. Therefore, this work focused on identifying and validating the potential ferroptosis-related genes involved in RA through bioinformatics analysis.

**Methods:**

We screened differentially expressed ferroptosis-related genes (DEFGs) between RA patients and healthy individuals based on GSE55235 dataset. Subsequently, correlation analysis, protein-protein interaction (PPI) network analysis, GO, and KEGG enrichment analyses were performed using these DEFGs. Finally, our results were validated by GSE12021 dataset.

**Results:**

We discovered 34 potential DEFGs in RA based on bioinformatics analysis. According to functional enrichment analysis, these genes were mainly enriched in HIF-1 signaling pathway, FoxO signaling pathway, and Ferroptosis pathway. Four genes (GABARPL1, DUSP1, JUN, and MAPK8) were validated to be downregulated by GSE12021 dataset and were diagnostic biomarkers and therapeutic targets for RA via the regulation of ferroptosis.

**Discussion:**

Our results help shed more light on the pathogenesis of RA. Ferroptosis-related genes in RA are valuable diagnostic biomarkers and they will be exploited clinically as therapeutic targets in the future.

## Introduction

Rheumatoid arthritis (RA) is a chronic autoimmune inflammatory disease characterized by inflammation and affects about 1% of the world population (1). As a result, RA can lead to joint deformity and disability, severely reducing the quality of life for RA patients. Although the pathogenesis of RA has not been entirely clarified, genetic and environmental factors have been shown to be involved (2). Several studies also demonstrated that the pathogenesis of RA was associated with certain biological functions such as osteoclast differentiation, inflammatory cell infiltration, angiogenesis, and ferroptosis (3, 4). Among these biological functions, ferroptosis is an interesting topic because of its potential links to cell death, suggesting a possible crucial role in the occurrence of RA.

Ferroptosis is a newly identified cell death form, which is characterized by iron-dependent accumulation and lipid peroxidation (5, 6). Evidence have reported that ferroptosis can trigger undesirable inflammatory responses (7, 8), and its inhibitor has an anti-inflammatory effect on different inflammatory diseases (9, 10). Various evidence has indicated that several pathways or compounds are related to ferroptosis in the pathogenesis of RA (4, 11, 12). Ferroptosis has been shown to be associated with RA, osteoarthritis, gout arthritis, and ankylosing spondylitis (13). However, the exact mechanism between ferroptosis and RA remains largely unclear, and it is necessary and urgent to elucidate the role in RA pathogenesis. The identification of potential ferroptosis-related genes in RA may help to derive potential biomarkers for treatment. Woetzel et al. (14) leveraged the GSE55235 dataset to obtain DEGs between RA patients and healthy individuals, their study sought to distinguish RA patients and healthy individuals by applying rule-based classifiers. However, they neglect the effect of ferroptosis on RA. Therefore, it is essential to analyze the relationship between ferroptosis and synovial inflammation, which may provide potential therapeutic targets for RA.

In this paper, we attempted to investigate the pathogenesis of RA from the ferroptosis perspective. To functionally identify and characterize the DEFGs in RA, we screened DEFGs based on the GSE55235 dataset and further validated our results in the GSE12021 dataset. Finally, this work may provide potential biomarkers for RA therapy and benefit the understanding of RA pathogenesis.

## Materials and methods

### Ferroptosis-associated genes and RA-related microarray dataset

The publicly available database FerrDb (15) (http://www.zhounan.org/ferrdb/) contains 259 ferroptosis-related genes. These downloaded data were used for subsequent analyses. Furthermore, we also collected gene expression profiles for 10 RA patients and 10 healthy individuals from the GSE55235 dataset to screen differentially expressed ferroptosis-related genes (DEFGs). The microarray data were quantified using the GPL96 platform from Affymetrix Human Genome U133A Array (14).

### Screening of DEFGs

First, we used the “limma” package to normalize the gene expression profile. Second, the probes were annotated using “annotation” package. We applied principal component analysis (PCA) to evaluate the repeatability of the GSE55235 dataset. The bioconductor “limma” package was subsequently utilized to screen DEFGs between RA patients and healthy individuals. |logFC| > 1 and adjusted *p*-value < 0.01 were set as the cutoff criteria of DEFGs. Lastly, we leveraged the “heatmap” and “ggplot2” packages to present the result of DEFGs.

### Functional enrichment analysis of DEFGs

Gene ontology (GO) is the widely used tool for gene function annotations. The GO terms are classified into three types: molecular function (MF), biological process (BP), and cellular component (CC). Pathway enrichment analysis was conducted using KEGG pathway database. The “clusterprofile” package (16) was utilized to perform the functional enrichment analyses of the DEFGs, with the cutoff criteria set at *p* < 0.01.

### PPI network construction and correlation analysis of DEFGs

The STRING database was used to schematically represent functional relationship network of DEFGs, named PPI network. We considered STRING interactions that were of medium confidence (combined STRING score > 0.4), which were experimentally derived and curated interactions. The PPI networks of DEFGs were visualized using the STRING database. Correlation analysis was performed using Pearson correlation coefficient with the “corrplot” package.

### Validation of gene expression associated with ferroptosis

The GSE12021 dataset was downloaded from the Gene Expression Omnibus (GEO) database, which includes 12 RA patients and 9 healthy individuals. The differentially expressed genes were also identified using the “limma” package according to the adjusted *p*-value < 0.01 and |logFC| > 1. Finally, we obtained the overlapping genes of 259 ferroptosis-associated genes, GSE55235 DEGs, and GSE12021 DEGs.

### Statistical analysis

R software (version 3.6.2) was used for statistical analysis. We applied the “limma” package to screen differentially expressed genes between RA patients and normal samples. The threshold was set at *p* < 0.01.

## Results

### Identification of 34 DEFGs in RA

**Figure 1:**
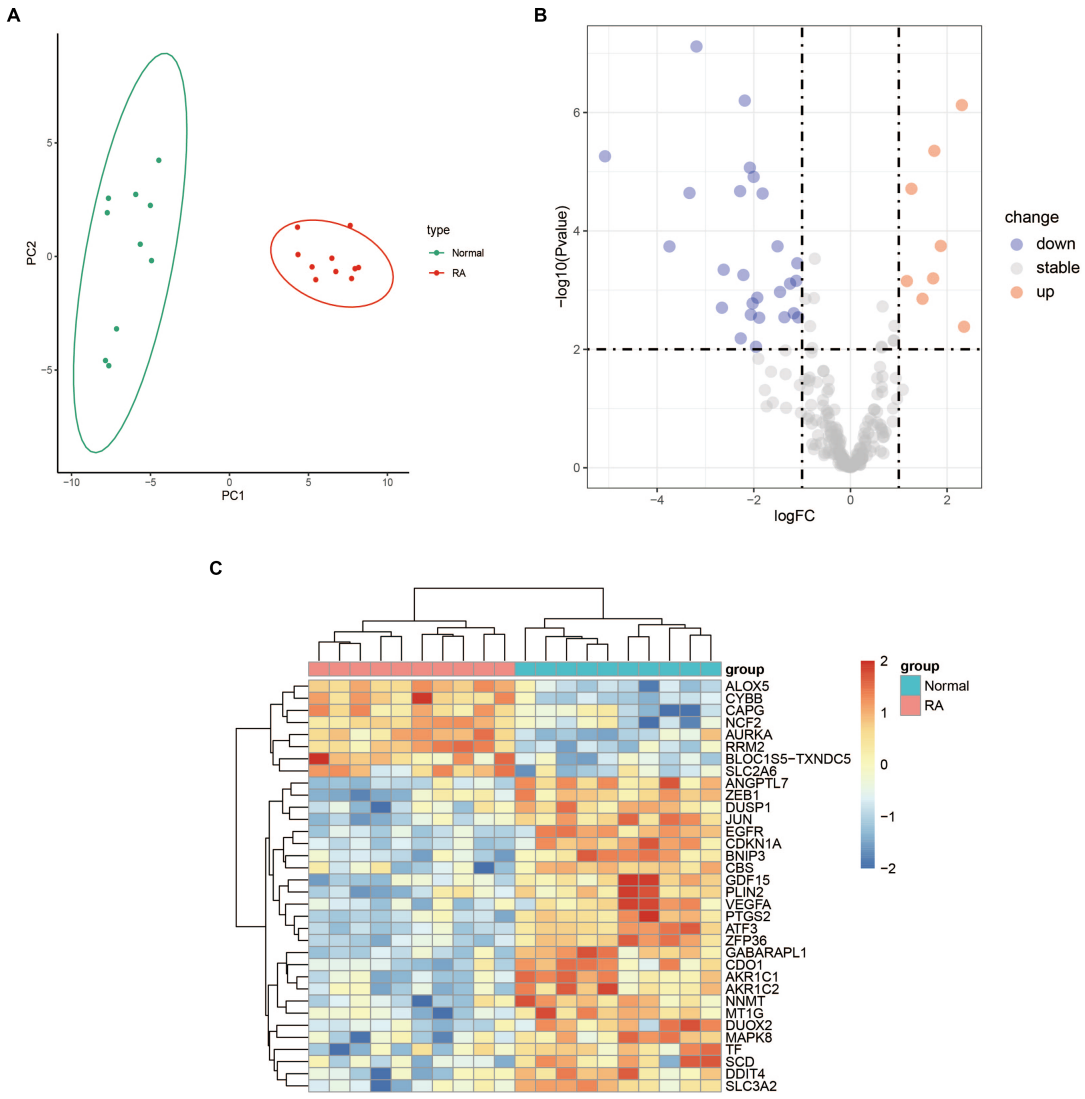
Differential expression analysis between RA patients and health samples. **(A)** PCA analysis of the GSE55235 dataset. **(B)** Volcano plot of the 259 ferroptosis-related genes. The significantly upregulated and downregulated genes are represented by red and blue dots, respectively. **(C)** Heatmap of the 34 DEFGs between the patients with rheumatoid arthritis and healthy individuals.

The result of PCA showed good biological replicate concordance of each biological sample in the GSE55235 dataset (Figure 1A). We integrated 259 ferroptosis-related genes and DEGs between RA patients and healthy individuals from the GSE55235 database to screen DEFGs. We obtained a total of 34 DEFGs, of which 8 were upregulated and 26 were downregulated genes (Figures 1B,C).

**Figure 2:**
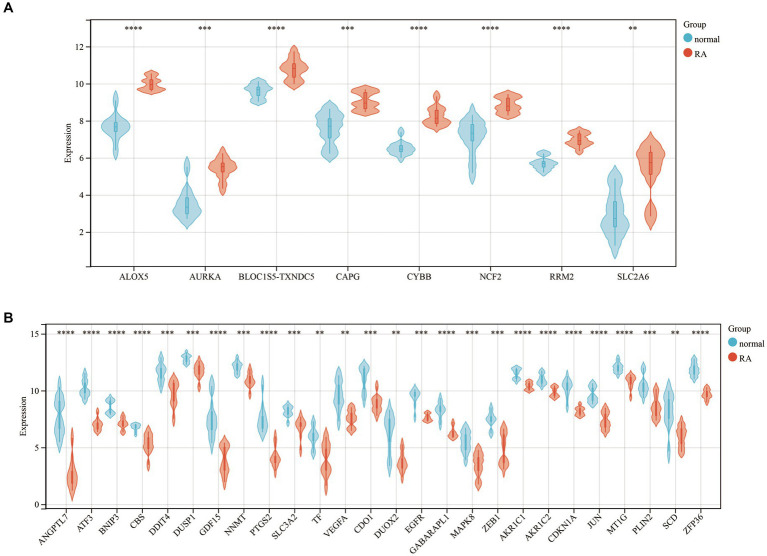
The violin diagram of 34 DEFGs between the patients with rheumatoid arthritis and healthy individuals. **(A)** 8 upregulated genes **(B)** 26 downregulated genes. **: *p*-value < 0.01, ***: *p*-value < 0.001, ****: *p*-value < 0.0001.

Furthermore, we generated a violin diagram to illustrate the expression patterns of the 34 DEFGs (Figure 2). There are 8 upregulated genes (Figure 2A) including SLC2A6, ALOX5, AURKA, CYBB, NCF2, CAPG, RRM2, BLOC1S5-TCNDC5, and 26 downregulated genes (Figure 2B) included ANGPTL7, ATF3, BNIP3, CBS, DDIT4, DUSP1, GDF15, NNMT, PTGS2, SLC3A2, TF, VEGFA, CDO1, DUOX2, EGFR, GABARPL1, MAPK8, ZEB1, AKR1C2, CDKN1A, JUN, MT1G, PLIN2, SCD, ZFP36.

### Construction of PPI network and correlation analysis of the 34 DEFGs

**Figure 3:**
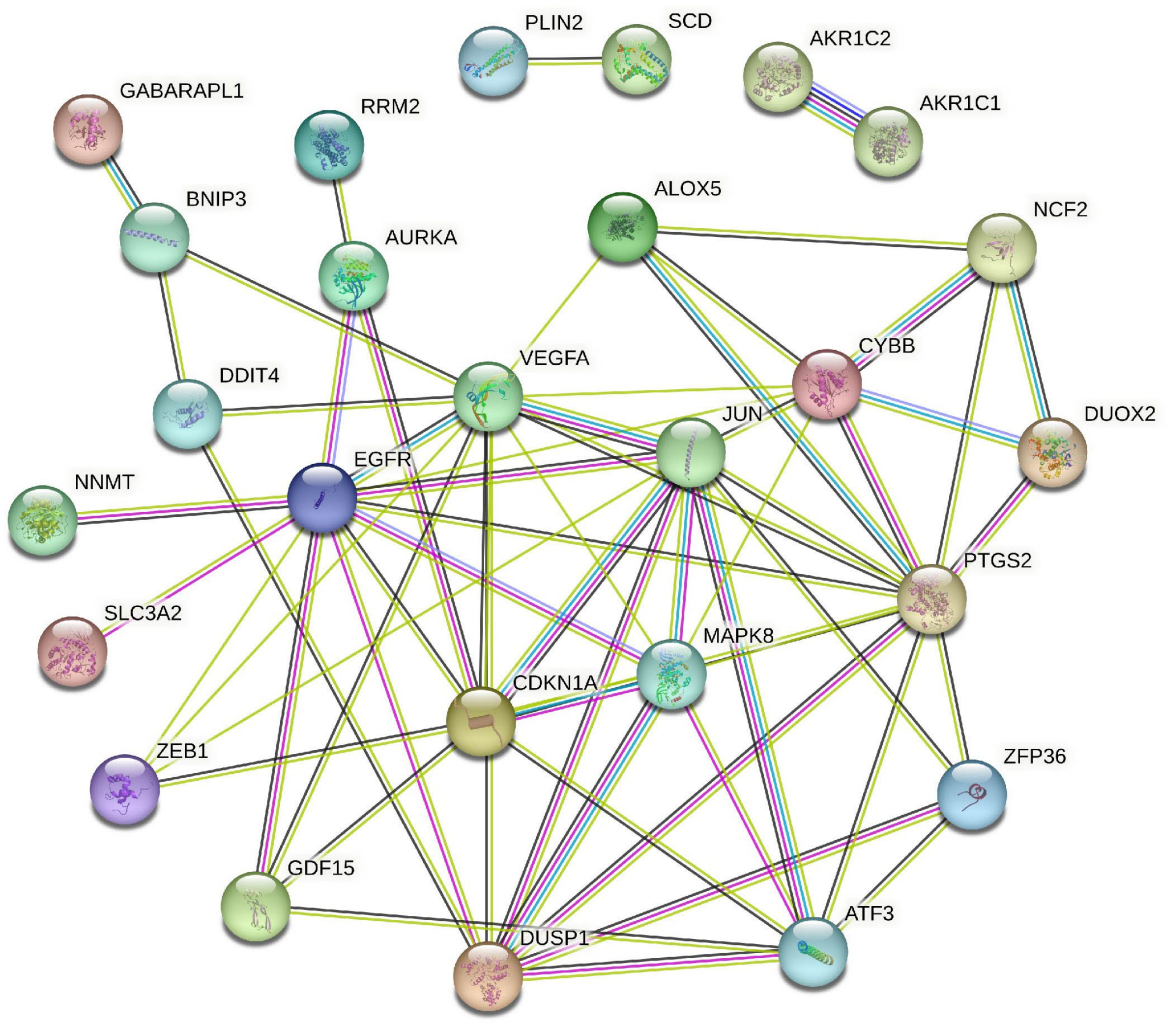
PPI network of 34 DEFGs.

**Figure 4:**
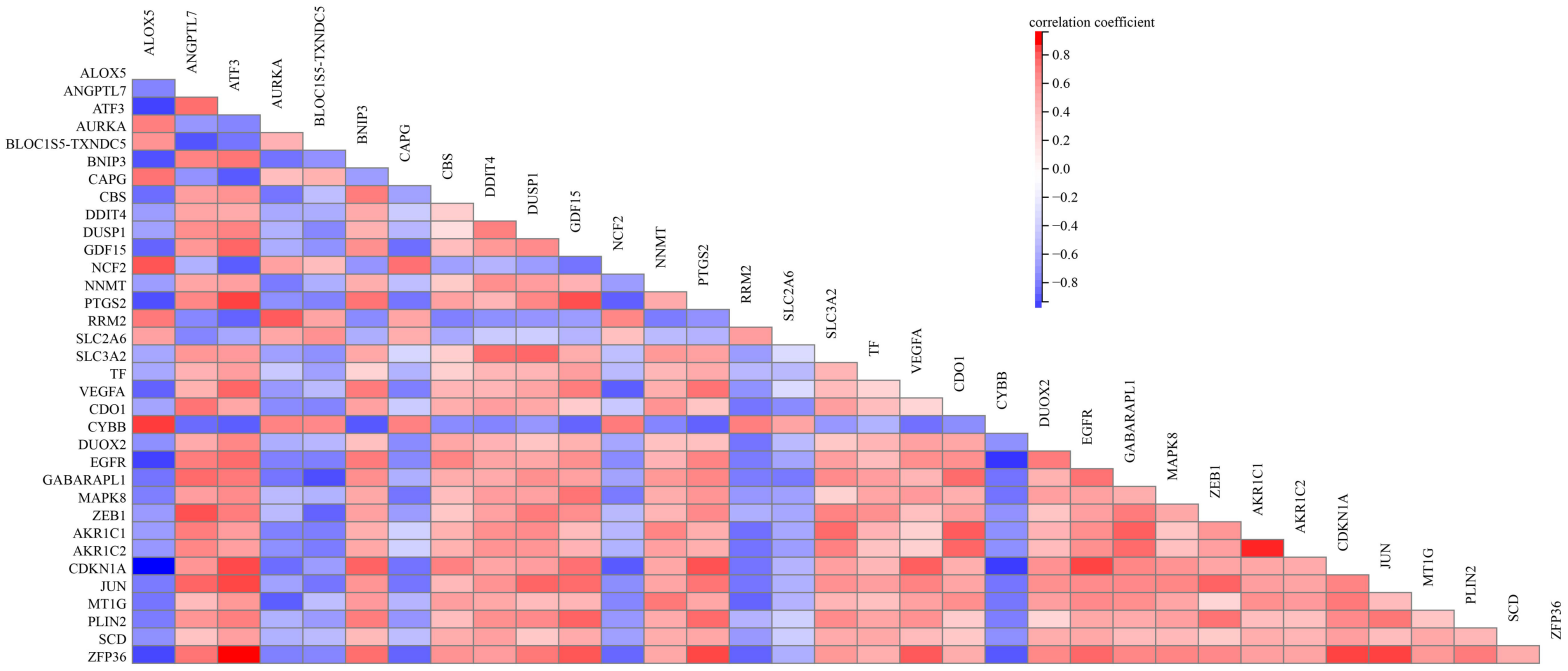
Pearson correlation analysis of the 34 DEFGs.

To elucidate the relationship of these 34 DEFGs, we performed PPI network analysis. The results demonstrated that 34 DEFGs were closely interconnected (Figure 3). We find several hub genes (VEGFA, JUN, CDKN1A, DUSP1, MAPK8, and EGFR) that have high degree. In addition, to examine if there was a correlation between the expression levels of these DEFGs, we conducted correlation analysis. The results showed that the correlation relationship of most genes is positive, only a few have negative correlation relationship (Figure 4).

### Functional enrichment analyses of the 34 DEFGs

**Figure 5:**
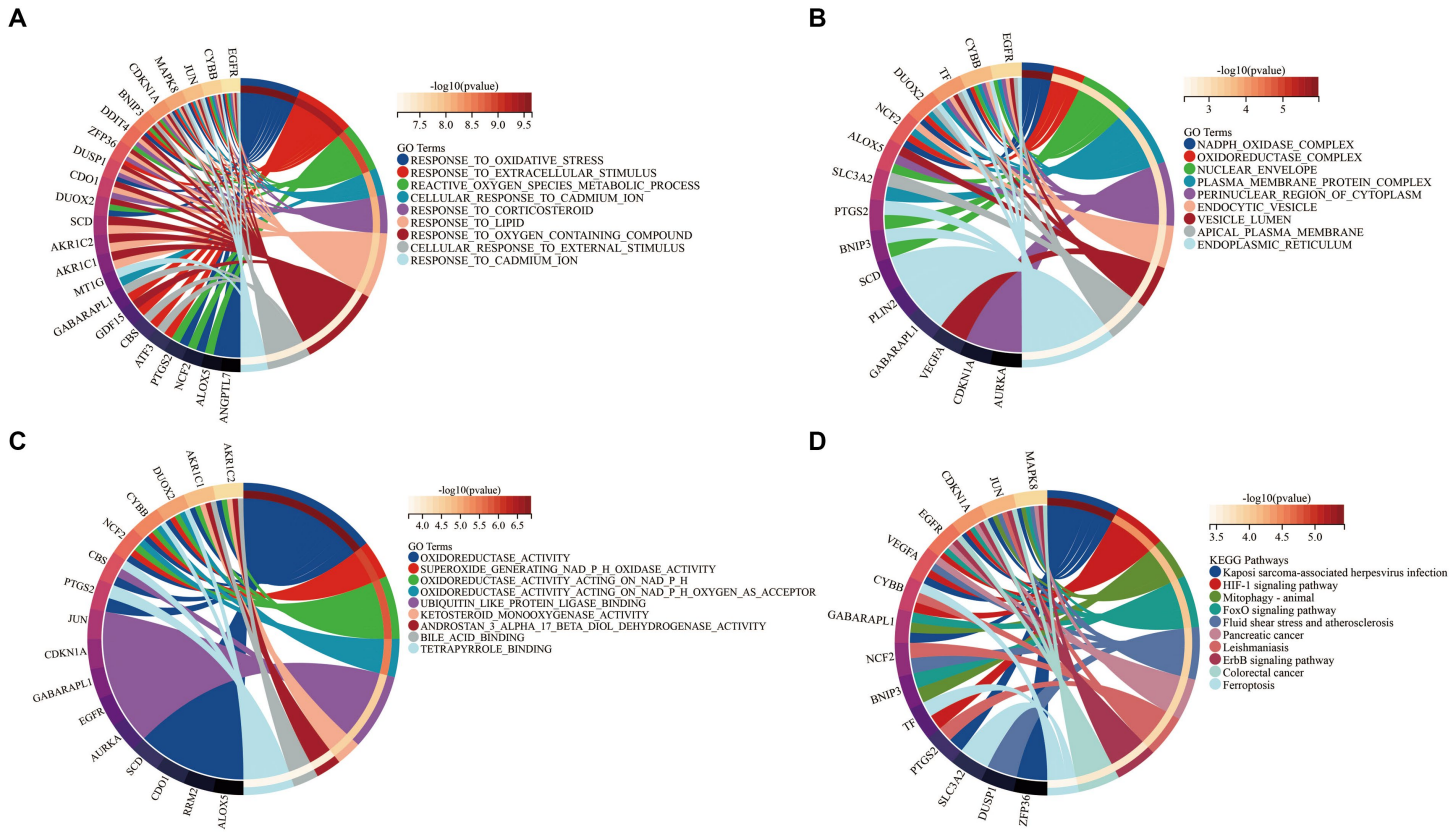
Functional enrichment analysis of 34 DEFGs. **(A)** BP, **(B)** CC, **(C)** MF, **(D)** KEGG. BP: biological process; CC: cellular component; MF: molecular function.

To elucidate the biological functions of the 34 DEFGs, GO and KEGG enrichment analyses were performed using the “clusterprofiler” package to identify significantly enriched terms. The most significantly enriched GO terms of BP were cellular response to oxidative stress, response to extracellular stimulus, and reactive oxygen species metabolic process (Figure 5A). In the CC category, they were involved in nicotinamide adenine dinucleotide phosphate (NADPH) oxidase complex, oxidoreductase complex, and nuclear envelope (Figure 5B). In the MF category, they were involved in oxidoreductase activity, superoxide generating NADPH oxidase activity, and oxidoreductase activity acting on nicotinamide adenine dinucleotide phosphate (NADPH; Figure 5C). KEGG enrichment was mainly distributed in HIF-1 signaling pathway, FoxO signaling pathway, and Ferroptosis pathway (Figure 5D).

### Validation of gene expression associated with ferroptosis

**Figure 6:**
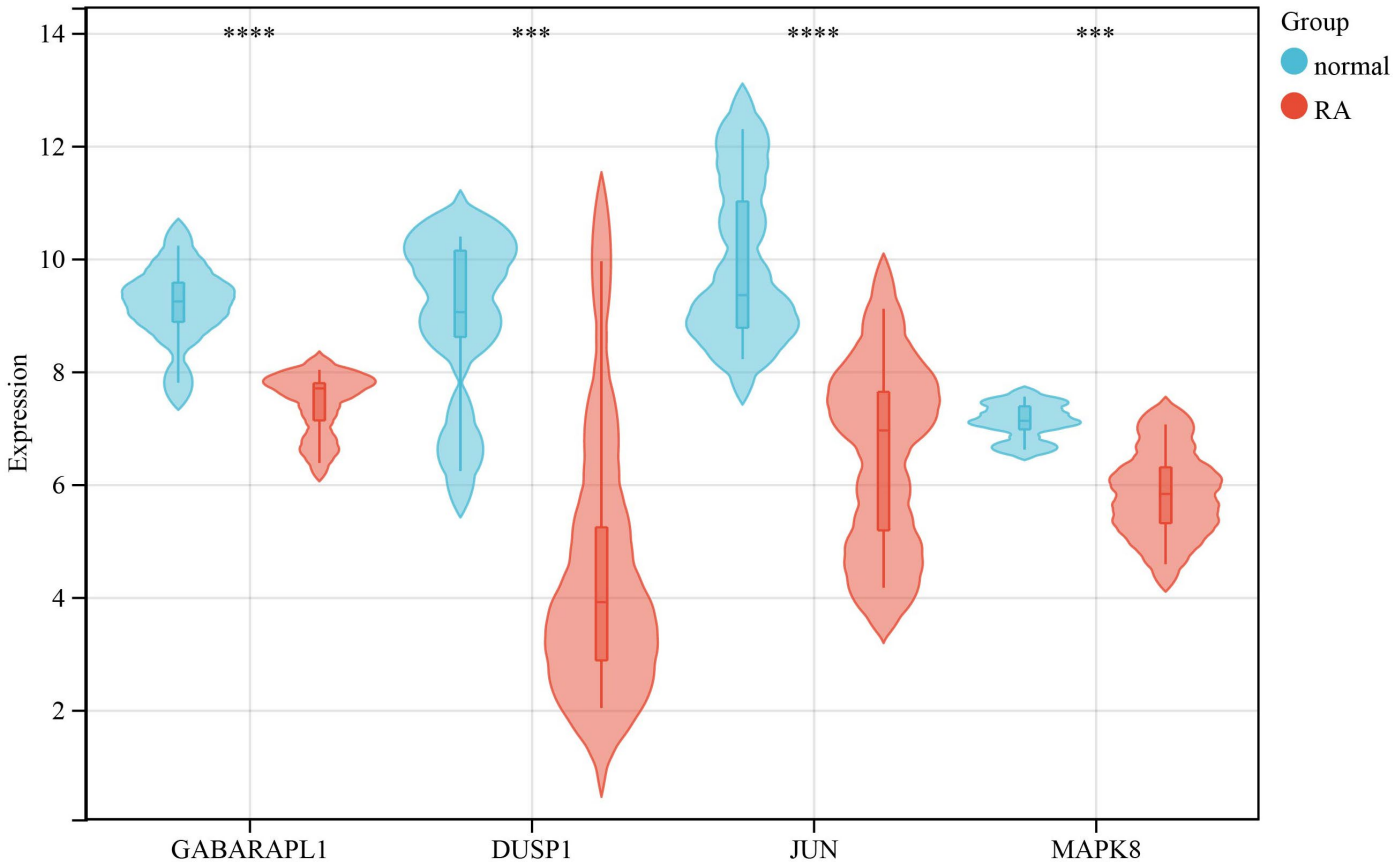
Validation of gene expression in the GSE12021 dataset. The violin diagram showed 4 DEFGs between RA patients and healthy individuals. The Wilcox test was used to compare RA patients with normal samples. ***: *p*-value < 0.001, ****: *p*-value < 0.0001.

To validate the reliability of 34 DEFGs expression levels, we used another dataset (GSE12021) (17) to verify expression levels. Compared with the results of the GSE55235 dataset, the expression levels of GABARPL1, DUSP1, JUN, and MAPK8 were decreased in the RA samples (−1.75-, −4.47-, −3.44-, and −1.28-fold, respectively) compared to normal samples (Figure 6). It indicated that the expression levels of four genes were consistent with the results of GSE55235.

### Prediction of potential therapeutic drugs

DGIbd (18) and CMAP database (19) were utilized to find potential candidate agents for GABARPL1, DUSP1, JUN and MAPK8. In the DGIbd or CMap database, Albuterol, Hydroxyurea, or Vasopressin was found to be the targeted medicine of DUSP1, Irisolidone, Holacanthone, Sergeolide or Irbesartan was found to be the targeted medicine of JUN, and Cardamomin, CC-401, Tanzisertib or BI-78D3 was found to be the targeted medicine of MAPK8 (Table 1). Ehrlich et al. (17) found that low-dose hydroxyurea held potential efficacy in the treatment of rheumatoid arthritis (RA). Petersson et al. (30) reported potential involvement of arginine-vasopressin and parathyroid hormone-related protein in the pathophysiological mechanisms of RA. Chang et al. (31) discovered that the angiotensin receptor blockers (ARBs) losartan and irbesartan effectively reduced superoxide levels, downregulated the expression and activity of NAD(P)H oxidases, and ameliorated endothelial dysfunction in antigen-induced arthritis (AIA). Zhou et al. (32) conducted a study in which they found that the addition of irbesartan to chemotherapy demonstrated potential for enhancing therapeutic effectiveness in patients with pancreatic ductal adenocarcinoma (PDAC) who exhibited elevated c-Jun expression levels. Irbesartan demonstrated significant efficacy in overcoming chemotherapy resistance by effectively suppressing the Hippo/YAP1/c-Jun/stemness/iron metabolism axis.

**Table 1:** Prediction of potential therapeutic drugs.

Drug	Gene	Source	PubChem	Ref
Albuterol	DUSP1	DGIdb	182176	(20)
Hydroxyurea	DUSP1	DGIdb	3657	(21)
Vasopressin	DUSP1	DGIdb	123131941	(22)
Irisolidone	JUN	DGIdb	5281781	(23)
Holacanthone	JUN	DGIdb	158475	(24)
Sergeolide	JUN	DGIdb	134025	(24)
Irbesartan	JUN	CMAP	3749	(25)
Cardamomin	MAPK8	DGIdb	641785	(26)
CC-401	MAPK8	DGIdb/CMAP	10430360	(27)
Tanzisertib	MAPK8	DGIdb	11597537	(28)
BI-78D3	MAPK8	CMAP	2747117	(29)

## Discussion

Although great progress has been made in exploring new treatments for RA. To date, elucidating the pathogenesis of RA remains a major challenge, thus it is urgent to explore the molecular mechanisms of these diseases. Moreover, several studies have illustrated that ferroptosis may be involved in various tumor types (33, 34). However, there have been few studies focused specifically on ferroptosis-associated genes in RA.

In our study, there were 34 potential ferroptosis-related genes that were identified between RA patients and healthy individuals by using bioinformatic analyses. Furthermore, GO and KEGG enrichment analyses were conducted to investigate the biological functions of the 34 DEFGs. The oxidative stress, oxidoreductase complex, and NADPH oxidase were significantly enriched by these DEFGs, indicating that these DEFGs were associated with oxidation, which was consistent with previous reports (35).

Based on KEGG pathway enrichment, we found that these genes were significantly enriched in FoxO signaling pathway, HIF-1 signaling pathway, and Ferroptosis pathway. Lee et al. (36) identified FoxO3 as a biomarker of RA severity, and its haplotype was associated with erosion scores of RA. Kok et al. (37) reported that SIRT-1/FoxO3a signaling played a crucial role in the occurrence and development of RA. Besides, FoxO signaling was also vital in OA. Abnormal expression of FoxO had been reported to be closely associated with osteoarthritis (38). HIF-1α is a very important transcription factor that regulates developmental and cellular responses to hypoxia. A deficiency of HIF-1α has been reported to exacerbate MMP13 expression levels to lead to degrading cartilage tissue (39). Our results represented these RA-related pathways, which might enhance our understanding of RA pathogenesis.

Furthermore, we identified 4 DEFGs (GABARPL1, DUSP1, JUN, and MAPK8) and observed that the expression levels of these genes in the GSE55235 were consistent with the GSE12021 dataset. Several genes had been shown to be closely related to RA. For example, DUSP1 is a phosphatase with dual specificity for tyrosine and threonine, which involves cellular processes by regulating MAPK1/ERK2. Vattakuzhi et al. (40) found that DUSP1 can regulate MAPK signaling and its low expression may be related to osteolytic lesions in arthritis. JUN is the putative transforming gene of avian sarcoma virus 17. It regulated gene expression by interacting with specific target DNA sequences (41). Huber et al. (42) revealed that Jun/Fos proto-oncogene was significantly decreased at the mRNA level in RA. MAPK8, also known as Jun nuclear kinase (JNK), was a member of the MAP kinase family, which was involved in various cellular processes (proliferation, differentiation, transcription regulation, and development) (43). Ding et al. (44) reported that abnormal activation of MAPKs in synovial tissues of RA patients promoted pannus formation. Thus, MAPK is considered a promising potential target in the treatment of RA. GABARAPL1, a constituent of the GABARAP family, demonstrates a remarkable degree of evolutionary conservation. Its coding gene was orignally identified as an early estrogen-induced gene (45). Gao et al. (46) uncovered that GABARAPL1 was a potential positive regulator of ferroptosis by RNAi screening. Furthermore, the expression of GABARAPL1 was notably diminished in various types of cancers (47–49). A comprehensive analysis of breast cancer biopsies in a cohort study revealed a significant association between lower GABARAPL1 expression and an increased risk of recurrence (48). Overexpression of GABARAPL1 exerted inhibitory effects on cell proliferation, colony formation, and invasion in breast cancer cells *in-vitro*. Xie et al. (50) demonstrated that DUSP1 inhibited ferroptosis by limiting lipid peroxidation while not affecting iron accumulation. Among the various mechanisms investigated in ferroptosis research, lipid peroxidation, which leads to the generation of lipid oxidation products, has garnered substantial attention. Cao et al. (51) discovered that the overexpression of JUN significantly suppressed the impact of T4O on both glioma cell proliferation and ferroptosis. Luo et al. (52) demonstrated that MAPK8 could reverse the impact of LINC01564 ablation on both cell apoptosis and ferroptosis.

However, this study still has some limitations. First, this work utilized a small sample size, which might lead to bias. Second, more *in-vivo* and *in-vitro* studies will be required to verify the reliability and significance of the results. Finally, it is necessary to understand the hub genes of RA for diagnosis and treatment.

In conclusion, we derived 34 potential ferroptosis-related genes between RA patients and healthy individuals using bioinformatics method. There were four genes (GABARPL1, DUSP1, JUN, and MAPK8) were verified to be differentially expressed and may serve as important diagnostic markers and new potential therapeutic targets for RA through the regulation of ferroptosis. Our study may be beneficial to enhance the understanding of RA pathogenesis and potential for clinical use in the future.

## Data availability statement

The original contributions presented in the study are included in the article/supplementary material, further inquiries can be directed to the corresponding authors.

## Author contributions

XL designed the study. XL and AH supervised the study and contributed equally to this work and should be regarded as co-first authors. XL, AH, YL, YH, and XZ analyze the data. XL and YL did the digital visualization and wrote and revised the manuscript. All authors contributed to the article and approved the submitted version.

## Funding

This work was supported by the National Natural Science Foundation of China (62202115), China Postdoctoral Science Foundation (2022M722746), and Zhejiang Province Natural Science Foundation (Q23H010006).

## Conflict of interest

The authors declare that the research was conducted in the absence of any commercial or financial relationships that could be construed as a potential conflict of interest.

## Publisher’s note

All claims expressed in this article are solely those of the authors and do not necessarily represent those of their affiliated organizations, or those of the publisher, the editors and the reviewers. Any product that may be evaluated in this article, or claim that may be made by its manufacturer, is not guaranteed or endorsed by the publisher.
